# Optimized two‐step electroporation process to achieve efficient nonviral‐mediated gene insertion into primary T cells

**DOI:** 10.1002/2211-5463.13292

**Published:** 2021-10-01

**Authors:** Ming Yang, Diane Tkach, Alex Boyne, Selena Kazancioglu, Aymeric Duclert, Laurent Poirot, Philippe Duchateau, Alexandre Juillerat

**Affiliations:** ^1^ Cellectis Inc New York NY USA; ^2^ Cellectis Paris France

**Keywords:** chimeric antigen receptors, double‐stranded break, gene editing, nonviral vectorization, TALEN

## Abstract

The development of gene editing technologies over the past years has allowed the precise and efficient insertion of transgenes into the genome of various cell types. Knock‐in approaches using homology‐directed repair and designer nucleases often rely on viral vectors, which can considerably impact the manufacturing cost and timeline of gene‐edited therapeutic products. An attractive alternative would be to use naked DNA as a repair template. However, such a strategy faces challenges such as cytotoxicity from double‐stranded DNA (dsDNA) to primary cells. Here, we sought to study the kinetics of transcription activator‐like effector nuclease (TALEN)‐mediated gene editing in primary T cells to improve nonviral gene knock‐in. Harnessing this knowledge, we developed a rapid and efficient gene insertion strategy based on either short single‐stranded oligonucleotides or large (2 Kb) linear naked dsDNA sequences. We demonstrated that a time‐controlled two‐step transfection protocol can substantially improve the efficiency of nonviral transgene integration in primary T cells. Using this approach, we achieved modification of up to ˜ 30% of T cells when inserting a chimeric antigen receptor (CAR) at the T‐cell receptor alpha constant region (*TRAC*) locus to generate ‘off‐the shelf’ CAR‐T cells.

## Graphical abstract

With understanding of TALEN‐mediated gene editing kinetics, we developed a rapid and efficient gene insertion strategy with naked DNA as template. We demonstrated that the time‐controlled two‐step transfection protocol can substantially improve the efficiency of nonviral transgene integration in primary T cells and achieved ˜ 30% integration frequency when inserting a 2 kb cassette into the T‐cell genome.

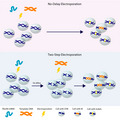

**Abbreviations**
AAV: adeno‐associated viruses
B2M: beta‐2 microglobulin
BGH PolyA: bovine growth hormone polyadenylation
CAR‐T cell: chimeric antigen receptor‐expressing T cell
CRISPR: clustered regularly interspaced short palindromic repeats
DSB: double‐stranded break
dsDNA: double‐stranded DNA
HDR: homology‐directed repair
IFNγ: interferon gamma
IL10: interleukin 10
IL6: interleukin 6
NHEJ: nonhomologous end‐joining
ssODN: single‐stranded oligodeoxynucleotides
TALEN: transcription activator-like effector nucleases
TCR: T-cell receptor
TNF-α: tumor necrosis factor-α
TRAC: T-cell receptor alpha constant region
ZFN: zinc finger nucleases

Cell‐based therapies have emerged in the past few years as a very successful approach to treat cancer and rare diseases. CAR‐T therapy, a targeted cellular immunotherapy that uses engineered T cells to specifically eliminate antigen‐bearing tumor cells, has been in the spot light achieving remarkable results in both preclinical and clinical applications for a variety of tumors [1]. Many methodologies have since been developed to optimize the performance of chimeric antigen receptor‐expressing T cell (CAR‐T cells), such as removing the T‐cell receptor (TCR) from healthy donor cells to generate universal CAR‐T cells [2]. Because universal CAR‐T cells are engineered out of third‐party healthy donor T cells, donors can be carefully chosen for potency and cells can be manufactured, formulated, and systematically quality controlled before being adoptively transferred to multiple patients in allogeneic settings. In addition, novel attributes are in test to enhance and control CAR‐T‐cell function [3, 4]. These novel approaches rely on our ability to efficiently and precisely control gene expression. Several strategies have been developed to allow for specific gene manipulations in tissue culture cells, colloquially known as ‘genome editing’. These strategies involve the use of molecular tools such as designer nuclease and base editors [5]. In particular, designer nucleases, including meganuclease, zinc finger nucleases (ZFN), transcription activator‐like effector nucleases (TALEN), and clustered regularly interspaced short palindromic repeats (CRISPR) nucleases can be engineered to create a double‐stranded break (DSB) at specific genomic target sequences [6].

In mammalian cells, two major pathways exist to repair DSBs—nonhomologous end‐joining (NHEJ) and homology‐directed repair (HDR) [7]. During NHEJ repair, the rejoining of broken DNA ends, using little or no sequence homology, involves the processing of ends such that nucleotides could be deleted or inserted at the break site prior to ligation and hence potentially create mutations and disruptions to the locus [8]. Scientists have long harnessed this process to create gene knock‐outs (KO) of functional genes in various cell types [9] including primary cells such as human T cells [10]. In contrast, the repair of a DSB through HDR requires substantial lengths of sequence homology so that a DNA end from one molecule can invade an homologous sequence and prime repair synthesis [8]. The HDR pathway has been used to introduce (‘Knock‐In’, KI) exogenous sequences at particular genomic loci allowing reprograming of cellular functions for therapeutical applications. Viral vectors such as adeno‐associated viruses (AAV) are widely used for many applications to deliver repair templates [3, 11, 12]. However, in the current GMP manufacturing landscape, the time and cost of AAV production can be a rate‐limiting step for many therapeutic developments [13]. Under this situation, increasing amount of effort is being made to deliver DNA repair templates using nonviral strategies [14]. Delivering so‐called naked DNA as repair template (single stranded or double stranded) is particularly attractive because of the ease of manufacturing. However, such DNA delivery methods are facing challenges such as vectorization efficiencies and toxicity [14, 15, 16], especially in primary cells [17]. To mitigate these limitations, several strategies such as incorporating chemical modifications into the DNA repair templates to prolong half‐life [18], increasing homology arm length [19], and manipulating cell cycle [20] have been investigated.

Here, we show that a better understanding of the cleavage kinetic of designer nucleases (demonstrated for TALEN) can help improve nuclease‐mediated gene knock‐in when using naked DNA as repair template. We found that the TALEN protein expression peaked around 20 h post‐transfection and diminished after 48 h. Concomitantly, unlinked DNA DSBs accumulated until around 20 h post‐TALEN transfection. Applying these findings, we sought to deliver naked DNA repair templates 20 h post‐TALEN transfection: the time when maximum number of cells have unlinked DSBs and are susceptible to DNA repair. We showed that this timed delivery of the DNA repair template (16‐ to 20‐h delay) is a simple and robust strategy to enhance nuclease‐based genome engineering, allowing substantial increase of targeted insertion rates of a short sequence (20 bp) as well as a large (2.3 kb) sequence containing a chimeric antigen receptor. We achieved up to 46% and 29% of targeted gene insertion, respectively. We demonstrate that this two‐step transfection strategy provides a flexible, efficient, rapid, and cost‐effective nonviral avenue to improve targeted genome reprograming of universal CAR‐T cells.

## Results and Discussion

### Kinetics of TALEN expression and DSB creation

**Fig. 1:**
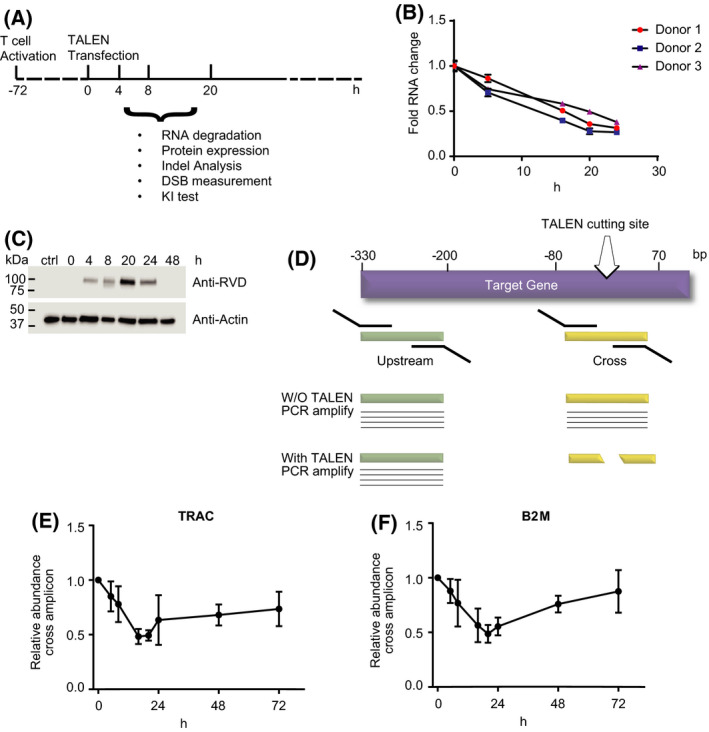
Kinetics of TALEN expression and DSB creation. (A) T‐cell activation and transfection protocol. Primary human PBMCs were thawed 1 day before CD3/CD28 bead activation. Three days postactivation, cells were de‐beaded and transfected with TALEN mRNA. The cells were then cultured for various length of time before being harvested for mRNA, DNA, or protein analysis. (B) Relative abundance of *TRAC* TALEN mRNA at different time points (15 min (0), 4, 16, 20, and 24 h) post‐transfection. The total RNA was extracted from cells from three different donors and subject to NGS‐based quantitation (*N* = 3 independent donors, 3 technical replicates, mean and SD). (C) The total cell lysate was extracted from nontransfected or TALEN mRNA‐transfected cells harvested at different time points. The cell lysate was resolved by SDS/PAGE and blotted with an anti‐RVD antibody, which detects the expression of the TALEN protein, or an antiactin antibody for loading control. (D) Scheme of qPCR strategy to measure unlinked DSBs created by the TALEN. (E) Fold change of unlinked DSBs at the *TRAC* TALEN target site (*N* = 3 independent donors, mean and SD). (F) Fold change of unlinked DSB at the *B2M* TALEN target site (*N* = 3 independent donors, mean and SD).

To understand the kinetics of TALEN expression, we transfected into primary human T cells an mRNA encoding a TALEN (*TRAC* TALEN) that targets the first exon of the T‐cell receptor alpha constant region (*TRAC*) gene [21]. The TALEN mRNA was vectorized using the BTX PulseAgile electroporation system, which was previously optimized for efficient delivery (gene editing frequencies and cell viability) of mRNA into primary T cells [3, 21, 22, 23]. Transfected cells were harvested at different timepoints up to 24 h postelectroporation, and the total mRNA was extracted for RNA‐seq analysis (Fig. 1A). Our results showed that the TALEN mRNA level gradually declined over time, reaching an average of 31% of its initial amount 24 h postelectroporation (Fig. 1B). The TALEN protein, produced from the transfected mRNA, was monitored in a separate experiment by western blot analysis. Our findings showed that the TALEN protein was detectable 4 h postelectroporation, accumulated until 20 h, and declined below detectable level at 48 h (Fig. 1C).

To quantitate the level of genomic DNA cleavage in response to the TALEN protein accumulation, we designed a qPCR assay to assess the relative abundance of intact genomic DNA at the *TRAC* TALEN target site. For this purpose, two sets of primer pair were used: The first set was designed to amplify a 164 bp amplicon encompassing a region from −102 to +62 bp (‘cross’ amplicon) across the *TRAC* TALEN cutting site which is defined as the center of the targeted sequence. For internal control, the second set was targeted to amplify a sequence from −292 to −120 bp (‘upstream’ amplicon) upstream of the *TRAC* TALEN cutting site (Fig. 1D) [24]. We expected the cross amplicon to inversely correlate with the abundance of TALEN‐driven unlinked DNA ends. Following TALEN mRNA electroporation, our data showed that the relative level of ‘cross’ amplicon decreased constantly, when normalized to the ‘upstream’ amplicon. The relative amount of ‘cross’ amplicon reached its lowest level 20 h postelectroporation (Fig. 1E), indicating that unlinked DSBs were most abundant 20 h post‐TALEN mRNA delivery. This finding was confirmed using a second TALEN targeting the first exon of the beta‐2 microglobulin gene (*B2M* TALEN). As previously, we quantified the abundance of a ‘cross’ amplicon that extended from −67 to +48 bp across the *B2M* TALEN cutting site and compared it to the abundance of a ‘downstream’ amplicon that spans from +112 to +323 bp downstream of the cut site. In accordance with the results obtained with the *TRAC* TALEN, this second data set showed that the largest portion of alleles harboring unlinked DNA ends at the TALEN target site also peaked at 20 h postelectroporation (Fig. 1F). As expected, the time point at which unlinked DSBs reached its highest proportion corresponded to the time when TALEN protein level peaked, showing the DNA cleavage event is directly related to the level of TALEN protein expression.

### Indel creation kinetics and Indel signature

**Fig. 2:**
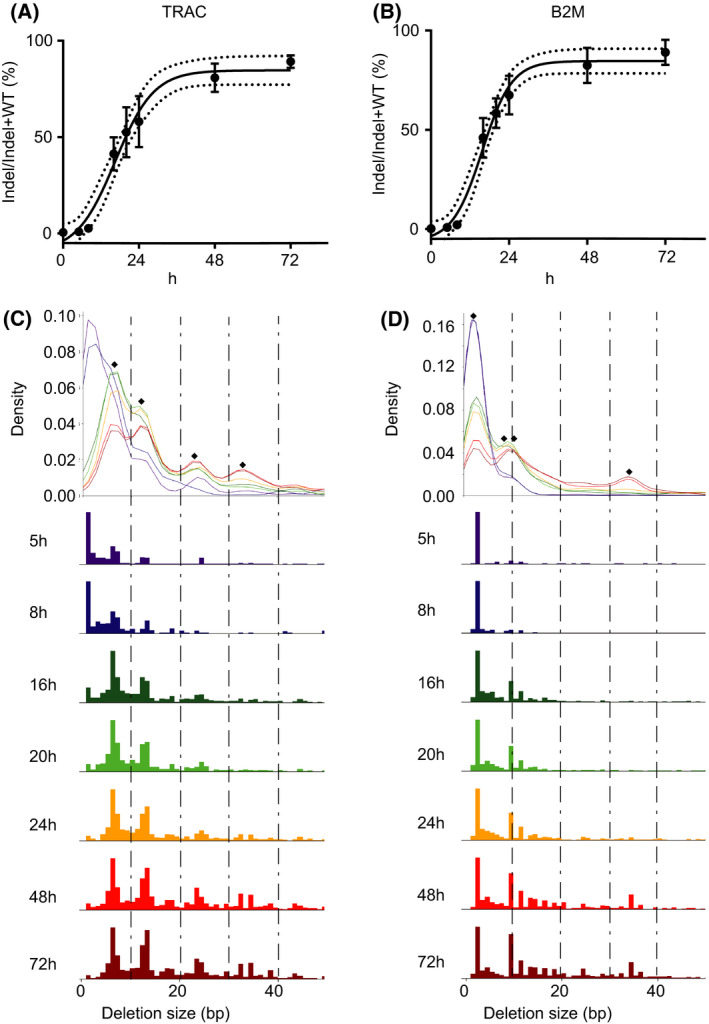
TALEN‐mediated Indel creation kinetics and Indel signature. (A, B) Time course experiment showing gradual accumulation of indels. Cells transfected with either *TRAC* or *B2M* TALEN mRNA were harvested at different time points after transfection for gDNA extraction. The genomic regions of TALEN target sites (*TRAC* or *B2M*) were PCR amplified and subjected to NGS analysis. A sigmoid curve was fitted to the data to show the indel accumulation. The dotted line represents the 95% confidence interval (*N* = 3 independent donors, mean and SD). (C, D) Size of deletions over time. The abundance of deletions at TALEN target sites was determined by NGS. Diamond points indicate enrichment of deletions with particular sizes/sequences (Table 1).

To better understand the different steps of TALEN genome editing, we then decided to further investigate the DNA repair events following the genomic DNA cleavage. Genomic DNA DSBs are mainly repaired through either the NHEJ or HDR pathways, although several others have also been described [5]. The NHEJ pathway, which could lead to the introduction of small insertions or deletions (indels), is usually favored over the more precise HDR pathway [25], especially when no exogenous HDR template is provided [26]. To further characterize the indel creation kinetics at the TALEN‐induced DSB sites (*TRAC* and *B2M* loci), we extracted genomic DNA from transfected cells at various timepoints up to 72 h post‐TALEN mRNA transfection. A region of 300‐bp across the TALEN target sites was amplified by PCR, and the resulting amplicons were subjected to next‐generation sequencing (NGS) to determine the percentage of indels. The results showed a rapid accumulation of indels over time, indicating that DSBs were created and eventually repaired with mutations (Fig. 2A,B). The indel frequencies reached plateau at 48 h post‐TALEN electroporation. For the *TRAC* TALEN, the plateau of indels frequency was 89.2% on average (SD: 3.2%), and for the *B2M* TALEN, the average was 89.0% (SD: 6.2%). The time to reach the indel plateau was in line with the kinetics of TALEN protein expression which fell below the detectable limit 48 h post‐transfection.

To further characterize the outcome of genomic DNA cleavage by the TALEN, we examined the pattern of deletion sizes over time. Our data showed that at early timepoints (< 8 h), with both *TRAC* and *B2M* TALEN, small deletions (< 5 bp) were the major species. At later time points, the abundance of small deletion decreased, and larger deletions started to accumulate (Fig. 2C,D). We have previously shown that, with the TALEN scaffold used in the current study, the TALEN activity was optimal on targets having 15 bp spacers (nucleotide sequence between the two DNA binding sequences of the two TALEN arms). The cleavage activity was strongly reduced when the spacer length decreases below 10 bp [27]. Thus, large deletions are likely to be a result of successive cleavage‐ligation cycles until the deletions are large enough to severely impact the TALEN cleavage. As expected, the indel size profile was stable between the 48‐h and 72‐h time points (Fig. 2C,D) which correlates with the disappearance of the TALEN protein 48 h post‐mRNA delivery (Fig. 1C). Interestingly, we also observed enrichment of specific deletion sizes in the *TRAC* TALEN (6, 12, 24, and 34 bp) and *B2M* TALEN (2, 9, 11, and 34 bp)‐treated samples. Detailed analysis of the genomic sequences flanking the deletions identified putative microhomology sequences (Fig. 2C,D and Table 1). These biases in deletion patterns were likely the outcome of the repair via the microhomology‐mediated end‐joining (MMEJ) mechanism [28].

**Table 1:** Sequences showing potential microhomology at the TALEN cutting site. ‘Deletion position’ is the position relative to the center of the spacer. In the Deletion Seq, lowercase lettering indicates deleted sequences while the intact flanking sequence (10 bp) is in uppercase lettering. The sequences underscored in red represent the deleted sequences that have homology with the rejoined sequences, which are in blue font.

TALEN target	Deletion position	Deletion size	Deletion Seq
TRAC	−3	6	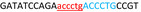
	−8	12	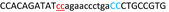
	−8	24	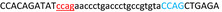
	−22	34	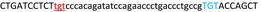
B2M	−2	2	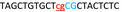
	−4	9	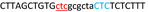
	−4	11	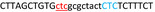
	−18	34	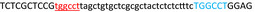

### Optimized gene modification with ssODN

In addition to NHEJ‐mediated DNA repair, DSBs can also be repaired via the HDR pathway. Cells can incorporate an exogenous sequence at DSB sites, especially when provided with DNA repair templates harboring homologies to the flaking sequences of the DSB sites. Single‐ and double‐stranded oligodeoxynucleotides (ssODN and dsODN) can be used as repair templates to introduce sequences into a genome [29]. Compared to double‐stranded DNA (dsDNA), ssODNs are less toxic and less inclined to insert randomly into the genome [30]. The target insertion of a short DNA sequence can be used to correct mutated genes [1] or to introduce stop codons [31] and epitope tags [24].

However, ssODNs have been shown to have a short (1.5 h) *in* *cellulo* half‐life postelectroporation [32], limiting their temporal availability to direct precise insertion via HDR. To overcome this limitation, we hypothesized that the optimal timing to deliver an ssODN repair template would be around the time when most of the cells have nuclease‐driven unlinked DNA breaks available for DNA repair.

**Fig. 3:**
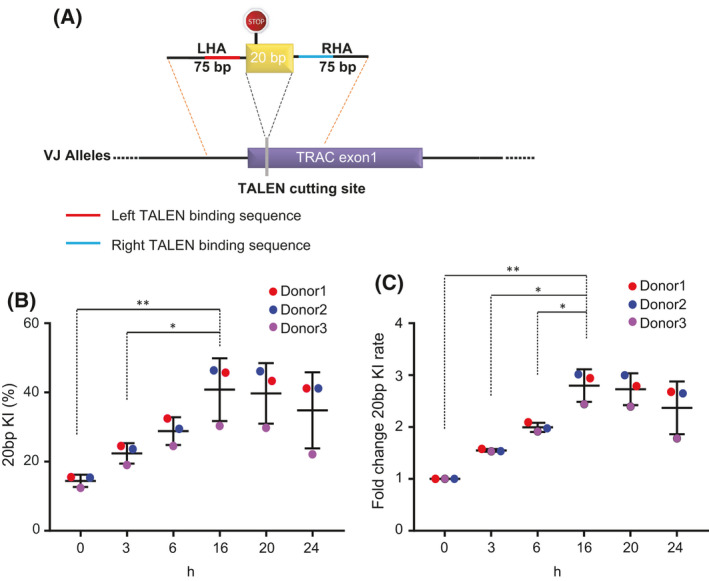
Two‐step electroporation improved gene modification with ssODN. (A) Design of the 20 bp insert ssODN. LHA: left homology arm, RHA: right homology arm. (B) ssODN KI frequencies measured by NGS (*N* = 3 independent donors, mean and SD). (C) Fold change in KI frequencies. KI frequencies were normalized within donors to the 0‐h time points. (*N* = 3 independent donors, mean and SD). (**P < 0.01, *P < 0.05, Student's *t*‐test).

To test this hypothesis, we used a 170 bp ssODN, containing a 20 bp exogenous sequence designed to insert a premature stop codon within the *TRAC* gene (at the TALEN cut site). The 20 bp sequence was flanked by 70 bp sequences homologous to the *TRAC* locus (homology arms, Fig. 3A). Following the TALEN mRNA transfection, cells were incubated for different lengths of time (0 h (15 min), 3, 6, 16, 20, and 24 h) before a second transfection for ssODN delivery (200 pmol ssODN per million cells). The ssODN was delivered with a second electroporator using a program adapted for DNA delivery [14]. Cells were harvested at Day 3 post‐TALEN transfection for NGS analysis to quantify ssODN insertion frequencies at the *TRAC* locus.

The results showed that the insertion frequency of 20 bp sequence significantly increased as we delayed the ssODN transfection (∼3‐fold, *P* = 0.0079 for 0 vs 16 h). We observed the maximum integration frequency (30% to 46%, mean = 40%, SD = 9%) at 16 h post‐TALEN electroporation (Fig. 3B,C). This observation confirmed our hypothesis that a timed delivery of the ssODN repair template can improve target insertion frequencies.

### Efficient nonviral CAR‐T‐cell engineering

**Fig. 4:**
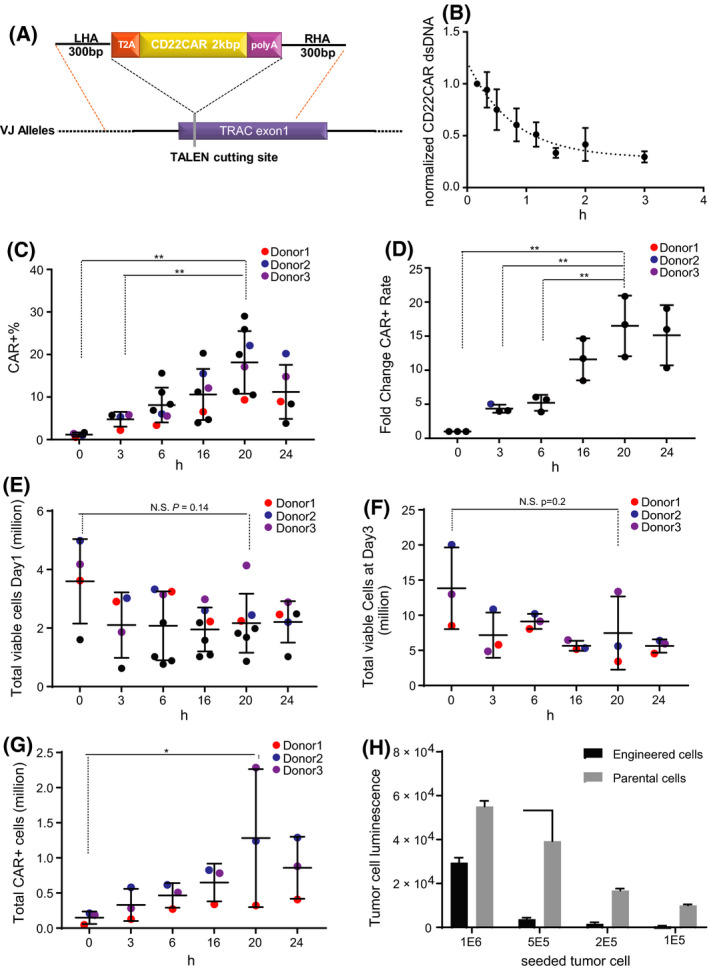
Two‐step electroporation improved CAR‐T cells engineering with dsDNA. (A) Scheme of the CD22CAR dsDNA repair template. (B) Half‐life of dsDNA repair template. Cells transfected with the CD22CAR dsDNA (PCR product) were harvested at different time points. qPCR was performed to quantify the amount of CD22CAR dsDNA within the cells. Data are normalized to 0 h. The curve was fitted using one phase decay. Half‐life = 54 min (*N* = 3 independent donors, mean and SD). (C) Total number of viable cells at Day 1 post‐dsDNA transfection. In one set of experiments, transfections were performed for three donors (blue, red, and purple dots) for all time points (*N* = 3–7 independent donors, mean and SD) (D) Percentage of CD22CAR expressing cells in the total cell population. In one set of experiments, transfections were performed for three donors (blue, red, and purple dots) for all time points (*N* = 3 to 7 independent donors, mean and SD). (E) Fold change in CAR+ frequencies. CAR+ frequencies were normalized within donors to the 0 h (*N* = 3 independent donors, mean and SD). (F) Total viable cells at Day 3 post‐dsDNA transfection (*N* = 3 independent donors, mean and SD). (G) Total number of CAR+ cells at Day 3 post‐dsDNA transfection. The total numbers of the CAR+ cell were calculated by multiplying the total number of viable cells (Fig. 4F) by the percentage of CAR+ cells in respective samples (Fig. 4D; *N* = 3 independent donors, mean and SD). (H) Luciferase assay showing the tumor cell luminescence after 24 h of incubation with T cells (engineered T cells or parental cells). Single donor in technical triplicates (mean and SD; **P < 0.01, *P < 0.05, Student's *t*‐test).

Because the length of ssODN (typically < 200 bp) limits its potential for large cDNA insertions, we explored whether our previous finding could be applied to the delivery of large (2.3 kb) coding gene (dsDNA). Toward this end, we designed a 2.3 kb dsDNA repair template aiming to insert a chimeric antigen receptor (anti‐CD22CAR) [3] at the *TRAC* TALEN target site. The CAR dsDNA repair template was designed to contain a ‘left’ *TRAC* homology arm (300 bp), a self‐cleaving P2A peptide coding sequence fused to a cDNA (1.4 kb) encoding the chimeric antigen receptor followed by a bovine growth hormone polyadenylation (BGH PolyA) signal (200 bp) and a ‘right’ *TRAC* homology arm (300 bp). When appropriately integrated at the *TRAC* locus, this promoterless DNA cassette will lead to the surface expression of a CD22CAR under the control of the endogenous *TRAC* gene regulatory elements (Fig. 4A). The DNA repair template was prepared by PCR amplification from a plasmid encoding the desired construct, which produced the linear dsDNA repair template (2.3 kb).

We first examined the half‐life of the dsDNA repair template in primary T cells. The cells, electroporated with the dsDNA, were harvested at different time points, and the abundance of the dsDNA was determined by qPCR. Consistent with observation from other groups [32], the qPCR analysis showed that the linear dsDNA template had a relatively short half‐life (T1/2 = 54 mins) *in* *cellulo* (Fig. 4B). As the dsDNA repair template showed a similar half‐life with reported half‐life of ssODN [32], we hypothesized that delaying the transfection of the long dsDNA repair template would also improve the integration efficiency.

To test this hypothesis, we transfected 5E6 of cells with 2 μg (2.3 pmol) of dsDNA template encoding the CD22CAR at different time points post‐*TRAC* TALEN mRNA transfection (Fig. 3B). The cells were then cultured for three more days before performing flow cytometry analysis to quantify the CD22CAR surface expression within the cell population. Our results demonstrated that transfecting the linear dsDNA repair template immediately after TALEN mRNA transfection led only to low frequencies of CAR surface expression (mean = 1.18%, SD = 0.49%; Fig. 4C). In contrast, the dsDNA transfection carried out 20 h post‐TALEN electroporation produced the highest CD22CAR surface expression, with a CAR+ cell population reaching up to 29% (mean = 18.16%, SD = 7.3%). These results demonstrated that delaying the dsDNA repair template transfection by 20 h significantly improved CAR+ population frequency by 16‐fold (SD = 4.5, *P* = 0.006; Fig. 4D) comparing to the 0‐h time point. We further noticed that, as expected, CAR+ cells were not present within the γδT‐cell population (Fig. S1) as the γδT cells do not express *TCRA* gene and the CD22CAR cassette was targeted at the *TCRA* locus and thus controlled by the expression of the *TCRA* gene.

Since the electroporation process, as well as the presence of exogenous polynucleotides (mRNA and naked DNA), might affect the cell viability and induce innate immune responses [29], we monitored cell number, growth, and the release of key cytokines postelectroporation. Day 1 post‐DNA electroporation, the 0‐h samples maintained an average of 3.5E6 viable cells from the 5E6 starting cells (SD = 1.4E6; Fig. 4E). In contrast, the 20‐h samples displayed a 40% reduction of the number of viable cells, with a mean of 2.1E6 viable cells (SD = 1.6E6). However, the decrease was not statistically significant (*P* = 0.14, 0‐h vs 20‐h samples). At Day 3 post‐DNA electroporation, the number of viable cells in 0‐h sample recovered to an average of 13E6 (SD = 5.8E6) representing a 3.7‐fold proliferation from Day 1 (Fig. 4F). The 20‐h sample cells recovered to an average of 7.5E6 (SD = 5.2E6), demonstrating proliferating capacities (3.5‐fold from Day 1) similar to the 0‐h samples. However, because the KI frequencies in the 20‐h delay sample were considerable higher (16‐fold; Fig. 4D) than for the 0‐h sample, we obtained a significant (*P* = 0.01) higher absolute number of CAR+ cells by delaying the CAR repair template transfection (0 h: 0.14E6, SD = 0.088E6; 20 h: 1.2E6 SD = 0.9E6; Fig. 4G).

In addition, at Day 2 post‐dsDNA transfection, we measured the release of cytokines (IL6, IL10, IFNγ, TNF‐α) from the cells undergoing the different transfection steps. We observed a general trend toward an increase in these cytokines with the different electroporation steps and in presence of mRNA and dsDNA (Fig. S2A). The secretion of TNF‐α increased significantly in the samples transfected with both the TALEN mRNA and the dsDNA, compared to the parental cells, suggesting that transfection steps indeed trigger a cellular immune response.

Three days post‐DNA electroporation, the samples from the 20‐h delayed dsDNA electroporation and the corresponding parental cells (no electroporation, no exogenous polynucleotides) were seeded in G‐REX gas permeable expansion devices for a large‐scale cell expansion. The cells were kept in G‐Rex for 13 days before harvest and cryopreservation. Edited cells from two independent donors were thawed and further analyzed for two key characteristics: the T‐cell differentiation (CD62L and CD45RA) and the exhaustion (LAG3 and PD1) marker profiles. We did not find major differences between the parental cells and edited cells (Fig. S2B,C).

In a separate experiment, we assessed the functionality of cells edited with the 20‐h delayed electroporation. Engineered cells (13% CAR+ cells at the end of 13 days of expansion process) or their parental un‐engineered control were incubated for 24 h with decreasing amounts of target Raji tumor cells (CD22+ and stably expressing luciferase). At the end of the co‐incubation, the remaining luciferase signal was used to assess the tumor cell survival. The result demonstrated that the engineered T cells promoted target cell killing, when compared to the matching parental un‐engineered T cells (Fig. 4H).

It is also worthy to mention that in our experimental conditions, ssODN and linear dsDNA do not require the same delivery time post‐TALEN mRNA electroporation to achieve the best performance and do not achieve similar integration frequencies. We assume that these differences are probably due to the amounts of DNA material (ssODN: 200 pm vs dsDNA: 2.3 pmol) we could deliver without causing significant level of toxicity. We also noticed that for both, short ssODN and long dsDNA, the integration frequencies declined slightly at their latest delivery timepoint (24 h), consistent with the decrease in TALEN protein which translates to lower DNA cleavage activity and thus reduced the loci availability for integration.

Nuclease‐driven targeted gene insertion offers great promise to precisely reprogram human cells, especially for adoptive cellular therapies. Within the last few years, viral vectors such as adeno‐associated virus (AAV) have been used to generate engineered T cells with a chimeric antigen receptor (CAR) genetically encoded at the endogenous *TRAC* locus (45%–65% of CAR+ cell within the population) [3, 11, 33]. More recently, nonviral (naked DNA) reprograming of T cells has been reported as an alternative to viral vectors. Targeted (*TRAC* locus) replacement of the endogenous TCR with an engineered TCR was explored (˜ 10% integration frequencies) [14, 34, 35]. Based on the understanding of nuclease‐driven genomic DNA cleavage and DSB repair kinetic parameters, we showed that a two‐step transfection process in which the nuclease mRNA is introduced first into the cells, followed by a second transfection (16–20 h later) of the repair template allows to significantly improve the nonviral targeted insertion of a short (20 bp, 3‐fold increase) and long (1.7 kb encoding a CAR, 16‐fold increase) sequence. Taken together, this study describes a simple and robust protocol to enhance nuclease‐based gene integration efficiency. We envision that this two‐step procedure could also be adapted to other designer nucleases such as ZFN or CRISPR/Cas9. However, depending on the format of the delivered nucleases (i.e., mRNA, RNP, or plasmid) and the kinetics of the nuclease expression and DSB creation, further optimization of the second transfection timing might be required. It was estimated in a Dox‐inducible Cas9 expression system, that the DSB formation peaked around 17 h after induction of the nuclease expression [36], a timing close to what we found with TALEN mRNA transfections. We anticipate that the KI efficiency in such system would also benefit from a delayed vectorization of repair templates. In contrast, if the CRISPR/Cas9 is to be delivered as an RNP complex, which would not require the nuclease expression step, creation of the DSB might be faster and a shorter delay of the second transfection should be considered.

The target insertion of a CAR at the *TRAC* locus strategy would not produce CAR‐expressing γδT cells. The γδT cells are thought to possess less allergenicity and more antitumor activity [37] and thus missing out on producing CAR γδT cells can be a drawback. Targeting the CAR to another locus that is expressed in both αβ and γδT cells could be a solution to this issue. Although the two‐step transfection protocol was performed in this study on primary T cells for universal CAR‐T‐cell generation, we anticipate that this strategy is broadly applicable to various cell types. Additional work, such as the evaluation of the level of random integration events [14], will nevertheless be required to fully assess the potential of this strategy before clinical applications. Taken together, our results expand the possibilities of nuclease‐mediated genome engineering in human primary cells and provide a foundation for further advances using nonviral gene editing methods.

## Materials and methods

### T‐cell culture

Cryopreserved human PBMCs were acquired from ALLCELLS. PBMCs were cultured in X‐vivo‐15 media (Lonza Group, Basel, Switzerland), containing 20 ng·mL^−1^ human IL‐2 (Miltenyi Biotech, Somerville, MA, USA), and 5% human serum AB (Seralab, New York, NY, USA). Human T‐cell activator CD3/CD28 Dynabeads (Thermo Fisher Scientific, Waltham, MA, USA) was used to activate T cells at 7 µL per million CD3+ cells.

### TALEN mRNA

Plasmids encoding the *TRAC* and *B2M* TALEN were produced in‐house. The TALEN mRNA was produced by *in vitro* transcription using the NEB HiScribe ARCA (NEB, Ipswich, MA, USA) kit. The sequences targeted by the two TALEN are the following (17‐bp recognition sites, upper case letters, separated by a 15‐bp spacer):

*TRAC:* (TTCCTCCTACTCACCATcagcctcctggttatGGTACAGGTAAGAGCAA).

*B2M:* (TCCGTGGCCTTAGCTGTgctcgcgctactcTCTCTTTCTGGCCTGGA).

### ssODN repair template

The ssODN targeting the TRAC locus (Table S1) was ordered from Integrated DNA Technologies (IDT, Coralville, IA, USA) and resuspended in ddH2O at 100 pmol·µL^−1^.

### Production of dsDNA repair template

A plasmid containing the CD22CAR repair template with sequences homologous to the *TRAC* target site was used as the PCR template [3]. Phosphorothioate‐modified primers (IDT) were used to amplify the target region (Table S1). PCRs were performed using the PrimeSTAR Max Premix (TaKaRa, Maebashi, Japan) system. In a 100 µL reaction, 40ng plasmid template and 4 µL of each of the 10 nm primers were used according to the manufacturer protocol. PCRs were set to 98 °C 10 s, 55 °C 5 s, 72 °C 12 s, 40 cycles. The PCR products were then purified with AMpure XP beads (1: 1 ratio; Beckman Coulter, Brea, CA, USA) and eluted into ddH_2_O.

### TALEN electroporation

T cells activated with CD3/CD28 beads (Life Technologies/Thermo Fisher Scientific) for 3 days were passaged into fresh complete media containing 20 ng·mL^−1^ human IL‐2 (Miltenyi Biotech) and 5% human serum AB (Seralab) 10‐12 h before transfection.

For TALEN mRNA transfection, the cells were first de‐beaded by magnetic separation with EasySep (Stemcell Technology, Cambridge, MA, USA), washed twice in Cytoporation buffer T (BTX Harvard Apparatus, Holliston, MA, USA), and 5E6 cells were then resuspended in 180 μL of Cytoporation buffer T. This cellular suspension was mixed with mRNA encoding TALEN at 1 µg mRNA per TALEN arm per million cells in a final volume of 200 µL. Transfection was performed using Pulse Agile technology by applying two 0.1 mS pulses at 3000 V·cm^−1^ followed by four 0.2 mS pulses at 325 V·cm^−1^ in 0.4‐cm gap cuvettes. The electroporated cells were then immediately transferred to a 12‐well plate containing 2 mL of prewarmed X‐vivo‐15 complete media and allowed to recover at 37 °C for 15 min before transfer to 30 °C.

### Target insertion with ssODN

For ssODN transfection, TALEN mRNA‐transfected cells were incubated at 30 °C for various length of time (15 mins (0), 4, 6, 16, 20, or 24 h) before harvesting. The harvested cells were washed once with warm PBS. 1E6 PBS‐washed cells were pelleted and resuspended in 20 µL Lonza P3 primary cell buffer (Lonza). 200 pmol ssODN repair template was mixed with the cell, and then, the cell mixture was electroporated using the Lonza 4D‐Nucleofector under the EO115 program for stimulated human T cells. After electroporation, 80 µL warm complete media was added to the cuvette to dilute the electroporation buffer, and the mixture was then carefully transferred to 400 mL prewarmed complete media in 48‐well plates. Finally, 5 unit·mL^−1^ benzonase (Sigma, St. Louis, MO, USA) was added to the cell culture to remove extracellular DNA. Cells transfected with ssODN were then incubated at 30 °C until 24 h post‐TALEN transfection before transfer back to 37 °C. 48 h after TALEN transfection, the cells were passaged to fresh complete media at 1E6 per ml.

### Target insertion of CD22CAR at the TRAC locus

For dsDNA transfection for target integration, the TALEN mRNA‐transfected cells were incubated at 30 °C for various length of time (15 min (0), 4, 6, 16, 20, or 24 h) before harvesting. The harvested cells were washed once with warm PBS. 5E6 PBS‐washed cells were resuspended in 100 µL Lonza Human T‐cell buffer (Lonza, 82 µL Human T‐cell buffer + 18 µL Supplement). Two micrograms dsDNA repair template (PCR product) was mixed with the cells, and the mixture was electroporated using the Lonza Nucleofector II under the T23 program for stimulated human T cells. After electroporation, 500 µL warm complete media was added to the cuvette to dilute the electroporation buffer, and the mixture was then carefully transferred to 2 mL of prewarmed complete media in 12‐well plates. Finally, 5 unit·mL^−1^ benzonase (Sigma) was added to the cell culture to remove extracellular DNA. Cells transfected with dsDNA were then incubated at 30 °C until 24 h post‐TALEN transfection before transfer back to 37 °C. 48 h after TALEN transfection, the cells were passaged to fresh complete media at 1E6 per mL.

### DNA extraction and qPCR

Cells were harvested and washed once with PBS. Genomic DNA extraction was performed using Mag‐Bind Blood & Tissue DNA HDQ kits (Omega Bio‐Tek, Norcross, GA, USA) following the manufacturer’s instructions.

For DSB detection, qPCR primers (Table S1) were designed to amplify the genomic sequence containing either the TALEN target sites or upstream (or downstream) of the TALEN target sites as controls. The qPCR was set up with the PowerUp SYBR Green Master Mix (Thermo Fisher) and analyzed on Bio‐Rad CFX (Bio‐Rad, Hercules, CA, USA). qPCR annealing temperature was 60 °C for all primers.

To determine the exogenous dsDNA half‐life, qPCR primers were designed to specifically amplify the CAR sequence. Primer pairs amplifying part of the genomic DNA of actin were used as loading control (Table S1). The qPCR was set up with the PowerUp SYBR Green Master Mix (Thermo Fisher) and analyzed on Bio‐Rad CFX. qPCR annealing temperature was 60 °C for all primers.

### Western blot analysis

To detect the expression of TALEN by western blots, cells were harvested at different time points after TALEN transfection. Cell lysate was prepared with 1x RIPA buffer (Millipore, Burlington, MA, USA) supplemented with 1x Halt Protease inhibitor cocktail (Thermo Scientific). Protein concentration was quantified with Pierce BCA protein assay (Thermo Scientific). 25 μg of total protein lysate from each sample was then resolved on an acrylamide gel (Bio‐Rad) and transferred to a nitrocellulose membrane. The expression of TALEN was detected by an anti‐RVD antibody (Table S2). An antiactin antibody (Table S2) was used to detect the level of actin in the lysate as loading control. The ECL (Thermo Scientific) signal was detected on Li‐COR (Li‐COR, Lincoln, NE, USA). See Table S2 for the list of antibodies used in this study.

### Flow cytometry

The information concerning antibodies used in this study is listed in Table S2. To detect the CD22CAR expression at the surface of the edited T cells, the cells transfected with TALEN mRNA and CD22CAR dsDNA were kept in culture for 96 h before harvesting and stained with a CD22Fc recombinant protein (Lake Pharma, Belmont, CA, USA) and an anti‐Fcγ secondary antibody. The CD3 antibody was used to detect the loss of CD3 as a result of *TRAC* knock‐out. Flow cytometry was performed on MACSQuant (Miltenyi Biotec) and data analysis processed with FlowJo. Cell population was first gated for lymphocytes (SSC‐A vs FSC‐A) and singlets (FSC‐H vs FSC‐A). The lymphocyte gate was further analyzed for expression of CD22CAR from this gated population.

To detect CD22CAR expression in γδT cells, the cells transfected with TALEN mRNA and CD22CAR dsDNA were kept in culture 3 additional days before harvesting and stained with a CD22Fc recombinant protein and TCR γδ antibody.

The cells transfected with the TALEN and CD22CAR dsDNA were cultured for 3 days after dsDNA transfection. At Day 3 post‐dsDNA transfection, the cells, as well as the untouched parental cells, were counted. 5E6 cells were seeded into a G‐Rex 10 gas permeable culture device (Wilson Wolf) for expansion in 40 ml of complete media. IL‐2 was replenished to the culture every 4 days to a final concentration of 20 ng·mL^−1^. 13 days after expansion, the cells were harvested and frozen in complete media with 10% DMSO in 10E6 cell aliquots.

Frozen vials of CAR‐T cells or parental cells were thawed and passaged in fresh complete media, at 1E6 per ml and kept in culture at 37 °C for 24 h before the measurement of T‐cell differentiation marker and exhaustion markers. To measure T‐cell exhaustion, cells were stained with anti‐PD1‐BV510 antibody and anti‐LAG3‐PerCP‐eFlour710 antibody. To measure T‐cell differentiation, cells were stained with an anti‐CD45RA‐VioBlue antibody (Miltenyi Biotec) and an anti‐CD62L‐PE antibody (Miltenyi) for flow cytometry analysis.

### CAR‐T‐cell activity assay

Parental cells or cells edited using the two‐step transfection protocol (20‐h delay) were seeded into G‐Rex10 for expansion for 13 days. Cells were then harvested. 1E6 T cells were then seeded with different amount of Raji cells (1E6, 5E5, 2E5, 1E5) which stably express luciferase in 24‐well plate. The cell mixtures were incubated at 37 °C for 24 h. Five microlitre cell suspension was collected and mixed with 25 μL of ONE‐glo buffer (Promega, Madison, WI, USA) for luciferase activity quantification.

### LEGENDplex cytokine releasing assay

Cells from different steps of the engineering procedure were cultured for an additional 48 h after the DNA transfection step. The cell culture supernatants were collected, and 15 μL of the supernatant was used in the LEGENDplex assay with BioLegend’s LEGENDplex Human T helper cytokine panel version 2 (BioLegend, San Diego, CA, USA) Cytokine release assay according to manufacturer’s protocol. The data were analyzed using BioLegend’s software Qognit.

### Deep‐sequencing indel analysis

At different time points after TALEN transfection, cells were harvested, washed once in PBS and subject to genomic DNA extraction by Mag‐Bind Blood & Tissue DNA HDQ kits (Omega Bio‐Tek).

For indel analysis, PCR amplifications spanning *TRAC* or *B2M* targets were performed using primers described in the Supplemental Materials. 1 μg genomic DNA was used per reaction in a 50 μL reaction with Phusion High‐Fidelity PCR Master Mix (NEB). The PCR condition was set to 1 cycle of 30 s at 98 °C; 30 cycles of 10 s at 98 °C, 30 s at 60 °C, 30 s at 72 °C; 1 cycle of 5 min at 72 °C; hold at 4 °C. The PCR product was then purified with Omega NGS beads (1 : 1.2 ratio) and eluted into 30 μL of 10 mm Tris buffer pH7.4. The second PCR which incorporates NGS indices to the sample was then performed on the purified product from the first PCR. 15 μL of the first PCR product was set in a 50 μL reaction with Phusion High‐Fidelity PCR Master Mix (NEB). The PCR condition was set to 1 cycle of 30 s at 98 °C; 8 cycles of 10 s at 98 °C, 30 s at 62 °C, 30 s at 72 °C; 1 cycle of 5 min at 72 °C; hold at 4 °C. Purified PCR products were sequenced on MiSeq (Illumina, San Diego, CA, USA) on a 2 x 250 Nano‐V2 cartridge. At least 150 000 sequences were obtained per PCR product, and sequences were analyzed for the presence of site‐specific indels.

### RNA‐seq

Cells were harvested at different time points after TALEN mRNA transfection. The RNA was extracted by TRIzol (Thermo Fisher). The total RNA QC was performed on Fragment Analyzer (Agilent, Santa Clara, CA, USA). PolyA pull‐down was then perform using NEBNext Poly(A) isolation module (NEB) to enrich for mRNAs. The RNA library was prepared with NEBNext Ultra II Directional RNA Library Prep Kit for Illumina (NEB) following manufacture’s instruction. The resulting RNA‐seq library was sequenced with Illumina NextSeq Hi‐Output cartridge. Data analysis was performed as follow: Read pairs were aligned using Bowtie 2 with local alignment on the N‐terminal sequence upstream of TALE repeat sequence, common to both TALEN arms. Reads mapping to the N‐terminal sequence were counted, and these counts were normalized to the number of total reads of the sample. Then, for each donor, normalized counts were re‐renormalized to the average value at time 0 for this donor.

### Statistical analysis

Unpaired Student’s *t* tests were used to analyze the data in Fig. 4C,E because the data points were collected from multiple independent experiments. Ratio paired Student’s *t*‐test was used to analyze the data presented in Fig. 3C,D, and Fig. 4D–G because the experiments were performed with three donors and the data for each time points were collected for each of the donors.

## Conflict of interest

All authors were Cellectis employees at the time of paper submission. TALEN® is a Cellectis' patented technology.

## Author contributions

MY, AJ, and PD designed experiments. MY and DT performed the experiments. AB designed ssODN oligos, and SK performed NGS sequencing. AD analyzed NGS data. MY AJ, PD, and LP analyzed the results.

## Supporting information


**Table S1**. Primers and Oligos.
**Table S2**. Information for antibodies used in this study.
**Fig. S1**. Expression of the CD22CAR in γδT cells. Six days after transfection, the expression of TCRαβ, TCRγδ and CD22CAR was measured by flow cytometry. To the right, the CD22CAR expression was gated on the TCRγδ+ population (red box). The TCRγδ− population (blue box) was further divided by their expression of CD22CAR and TCRαβ.
**Fig. S2**. T cell activation, differentiation and exhaustion after the two‐step electroporation. (A) Cytokine (IL6, IL10, IFNγ and TNFα) profile after electroporation of mRNA and/or dsDNA. Cells were cultured for 2 additional days before supernatant collection and analysis of cytokine expression by LEGENDPlex (*N* = 3 independent donors, median and range). (B) Expression of T cell differentiation markers after G‐Rex for expansion and cryopreservation. Thawed cells from two donors were culture for 24 h before staining for CD62L and CD45RA (*N* = 2 independent donors). (C) The same cells were stained for LAG3 and PD1 to detect the exhaustion markers (*N* = 2 independent donors).

## Data Availability

All data generated or analyzed during this study are included in the article.
