# Supplementary material for: Optimized two‐step electroporation process to achieve efficient nonviral‐mediated gene insertion into primary T cells
Source: FEBS Open Bio. 2021 Oct 1;12(1):38–50. doi: 10.1002/2211-5463.13292 (PMC8727936; doi:10.1002/2211-5463.13292)
Supplement: Supplementary file 1 — Table S1. Primers and Oligos. Table S2. Information for antibodies used in this study. Fig. S1. Expression of the CD22CAR in γδT cells. Six days after transfection, the expression of TCRαβ, TCRγδ and CD22CAR was measured by flow cytometry. To the right, the CD22CAR expression was gated on the TCRγδ+ population (red box). The TCRγδ− population (blue box) was further divided by their expression of CD22CAR and TCRαβ. Fig. S2. T cell activation, differentiation and exhaustion after the two‐step electroporation. (A) Cytokine (IL6, IL10, IFNγ and TNFα) profile after electroporation of mRNA and/or dsDNA. Cells were cultured for 2 additional days before supernatant collection and analysis of cytokine expression by LEGENDPlex (N = 3 independent donors, median and range). (B) Expression of T cell differentiation markers after G‐Rex for expansion and cryopreservation. Thawed cells from two donors were culture for 24 h before staining for CD62L and CD45RA (N = 2 independent donors). (C) The same cells were stained for LAG3 and PD1 to detect the exhaustion markers (N = 2 independent donors). [file FEB4-12-38-s001.pdf]

Supporting information

Table S1. Primers and Oligos

DSB primers	**B2M down_L**	CATTCCTGAAGCTGACAGCATTCGGG
	**B2M down_R**	GGGTAGGAGAGACTCACGCTGGATAG
	**B2M cross_L**	CGTGACTTCCCTTCTCCAAGTTCTCC
	**B2M cross_R**	ACGCTTATCGACGCCCTAAACTTTGT
	**TRAC up_L**	GCATTTCAGGTTTCCTTGAGTGGCAG
	**TRAC up_R**	TGGCAAGTCACGGTCTCATGCTTTAT
	**TRAC cross_L**	CTTGTCCATCACTGGCATCTGGACTC
	**TRAC cross_R**	ATCGGTGAATAGGCAGACAGACTTGT
qPCR primers for dsDNA	**Actin_L**	GCTGTTCCAGGCTCTGTTCC
Half-life	**Actin_R**	ATGCTCACACGCCACAACATGC
	**CAR_L**	GAATTGGATCAGGCAGTCCCCTTCTC
	**CAR_R**	TGTGATTCTGCTCTTCACAGACACGG
NGS primers	**B2M_L**	CTACACGACGCTCTTCCGATCTGTCCCTCTCTCTAACCTGGC
	**B2M_R**	GTGACTGGAGTTCAGACGTGTGCTCTTCCGATCTGAAGGGAAGTCACGGAGCGA
	**TRAC_L**	GTCGACTAGGGATAACAGGGTAATTATCCAGAACCCTGACCCTGCCGTGTACCA
	**TRAC_R**	AAACTGTATTATAAGTAAATGCATTGGATTTAGAGTCTCTCAGCTGGTACACGG
ssODN		CCTGGGTTGGGGCAAAGAGGGAAATGAGATCATGTCCTAACCCTGATCCTCTTG TCCCACAGATATCCAGAACCCTAGGTGAAAGCTTAGACTAGTGACCCTGCCGTGT ACCAGCTGAGAGACTCTAAATCCAGTGACAAGTCTGTCTGCCTATTCACCGATTTTGATTC
CAR template primers	**Template_L**	C*C*C*A*GTCACGACGTTGTAAAACG
	**Template_R**	G*C*G*GATAACAATTTCACACAGG

* phosphorothoiate bond

Table S2. Information for antibodies used in this study

Target	*Fluorophore*	*Company*	*Catalog #*	*Clone*	*Dilution*
Anti-rabbit IgG, HRP		Cell Signaling Technology	7074		1:10000
Actin		Sigma	A3853	AC-40, monoclonal	1:1000
Anti-mouse IgG, HRP		Cell Signaling Technology	7076		1:10000
CD22Fc recombinant protein		Lake Pharma			1:200
Goat Anti-Mouse IgG (subclasses 1+2a+2b+3), Fcγ	PE	Jackson Lab	115-115-164	polyclonal	1:200
Goat Anti-Mouse IgG, Fcγ subclass 1 specific	BV421	Jackson Lab	115-675-205	polyclonal	1:300
CD3	APC	Miltenyi	130-113-125	BW264/56	1:20
TCR-gd	PEVio770	Miltenyi	130-113-505	11F2	1:20

PD1	BV510	BD Biosciences	563076	EH12.1	1:25
LAG3	PerCP-eFlour710	Invitrogen	46-2239-42	3DS223H	1:25
CD45RA	Vioblue	Miltenyi	130-113-360	T6D11	1:25
CD62L	PE	Miltenyi	130-113-625	REA615	1:25

Supplementary Figure Legends

Figure S1. Expression of the CD22CAR in γδT cells. Six days after transfection, the expression of TCRαβ, TCRγδ and CD22CAR was measured by flow cytometry. To the right, the CD22CAR expression was gated on the TCRγδ+ population (red box). The TCRγδ- population (blue box) was further divided by their expression of CD22CAR and TCRαβ.

Figure S2. T cell activation, differentiation and exhaustion after the two-step electroporation. A. Cytokine (IL6, IL10, IFNγ and TNFα) profile after electroporation of mRNA and/or dsDNA. Cells were cultured for 2 additional days before supernatant collection and analysis of cytokine expression by LEGENDPlex (N=3 independent donors, median and range). B. Expression of T cell differentiation markers after G-Rex for expansion and cryopreservation. Thawed cells from two donors were culture for 24hrs before staining for CD62L and CD45RA (N=2 independent donors). C. The same cells were stained for LAG3 and PD1 to detect the exhaustion markers (N=2 independent donors).

Figure S1

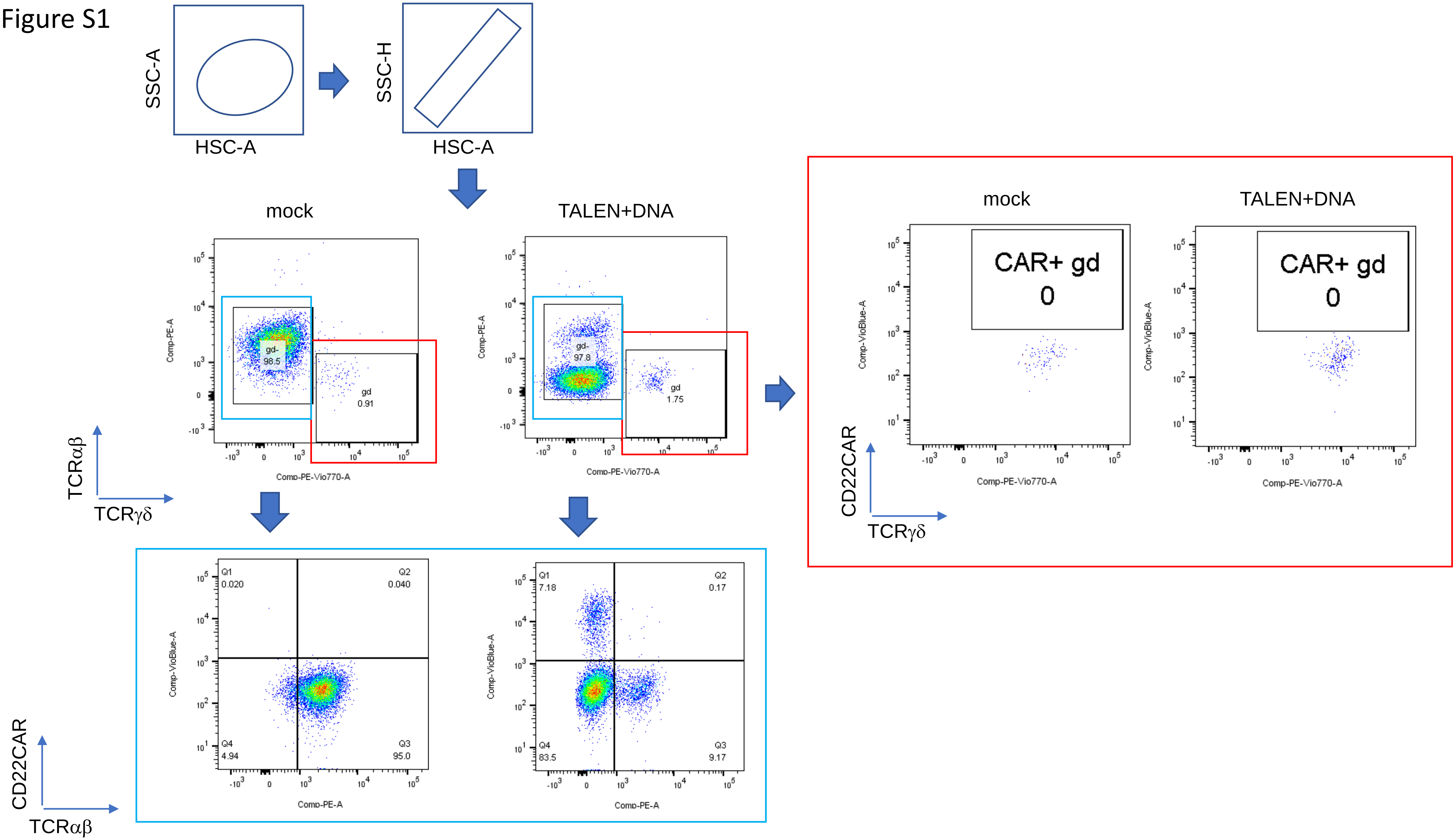
SSC-A
HSC-A
SSC-H
HSC-A
mock
TALEN+DNA
gd-
98.5
gd
0.91
gd-
97.8
gd
1.75
Comp-PE-A
Comp-PE-Vio770-A
TCRαβ
TCRγδ
mock
TALEN+DNA
CAR+ gd
0
CAR+ gd
0
Comp-VioBlue-A
Comp-PE-Vio770-A
CD22CAR
TCRγδ
Q1
0.020
Q2
0.040
Q4
4.94
Q3
95.0
Q1
7.18
Q2
0.17
Q4
83.5
Q3
9.17
Comp-VioBlue-A
Comp-PE-A
CD22CAR
TCRαβ

Figure S2

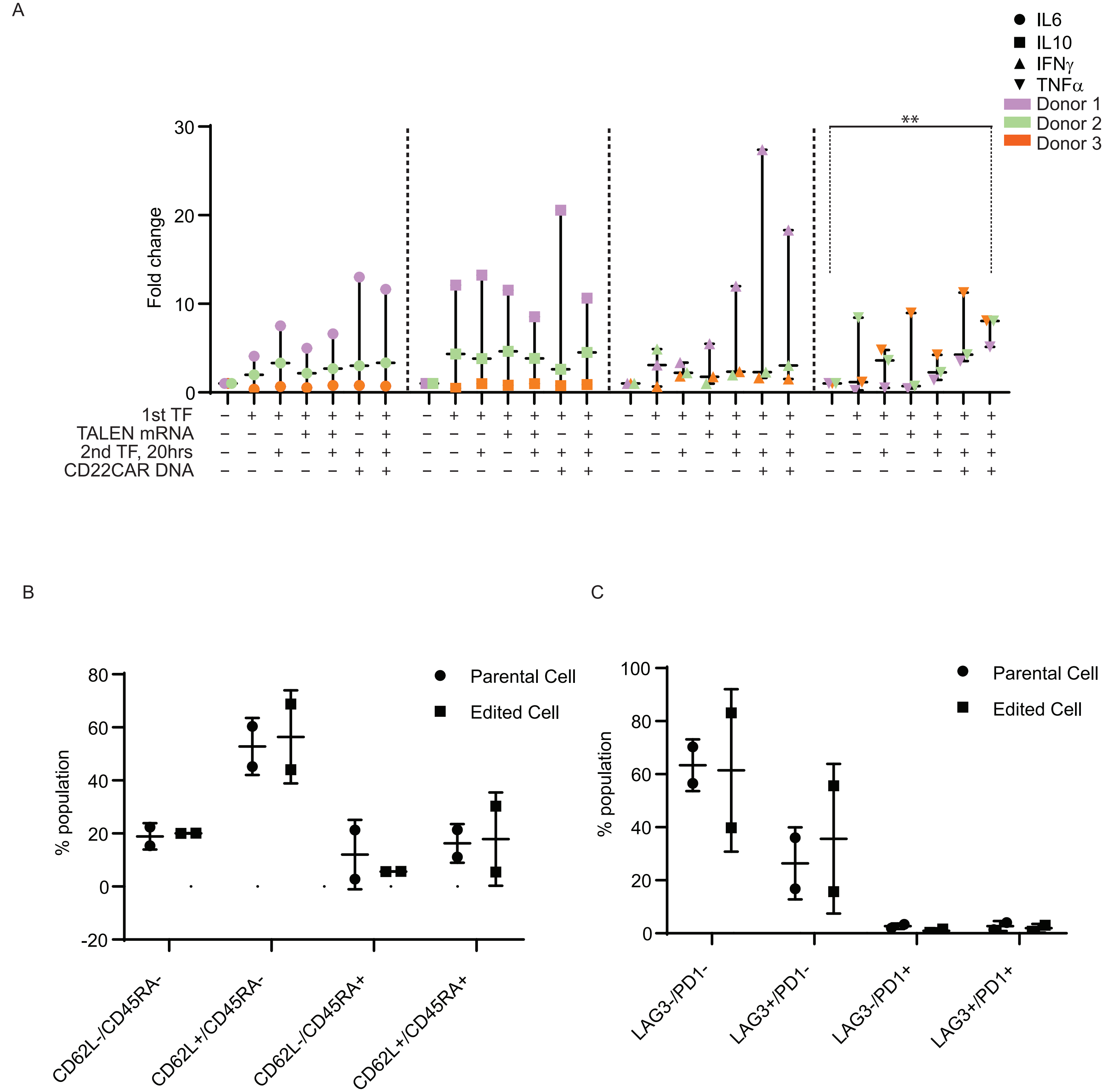
A
IL6
IL10
IFNγ
TNFα
Donor 1
Donor 2
Donor 3
**
Fold change
30
20
10
0
1st TF
TALEN mRNA
2nd TF, 20hrs
CD22CAR DNA
B
% population
80
60
40
20
0
-20
Parental Cell
Edited Cell
CD62L-/CD45RA-
CD62L+/CD45RA-
CD62L-/CD45RA+
CD62L+/CD45RA+
C
% population
100
80
60
40
20
0
Parental Cell
Edited Cell
LAG3-/PD1-
LAG3+/PD1-
LAG3-/PD1+
LAG3+/PD1+
